# Perspectives on Exercise Intensity, Volume and Energy Expenditure in Habitual Cycle Commuting

**DOI:** 10.3389/fspor.2020.00065

**Published:** 2020-06-30

**Authors:** Peter Schantz, Jane Salier Eriksson, Hans Rosdahl

**Affiliations:** Research Unit for Movement, Health and Environment, The Åstrand Laboratory and Laboratory of Applied Sport Science, The Swedish School of Sport and Health Sciences, GIH, Stockholm, Sweden

**Keywords:** commuter cycling, exercise intensity, maximal oxygen uptake, ventilation, energy expenditure, metabolic equivalent of task, trip duration, trip frequency

## Abstract

**Background:** Knowledge about exercise intensity and energy expenditure combined with trip frequency and duration is necessary for interpreting the character and potential influencing capacity of habitual cycle commuting on e.g., health outcomes. It needs to be investigated with validated methods, which is the purpose of this study.

**Methods:** Ten male and 10 female middle-aged habitual commuter cyclists were studied at rest and with maximal exercise tests on a cycle ergometer and a treadmill in the laboratory. During their normal commute in the Stockholm County, Sweden, their oxygen uptake, heart rate, energy expenditure, ventilation, blood lactate, rated perceived exertion, number of stops, durations, route distances and cycling velocities were monitored with validated methods. The frequency of trips was self-reported.

**Results:** The relative exercise intensity was 65% of maximal oxygen uptake, and the energy expenditure was 0.46 kcal per km and kg body weight for both sexes. Sex differences in MET-values (men, 8.7; women 7.4) mirrored higher levels of cycling speed (20%), body weight (29%), oxygen uptake (54%) and ventilation (51%) in men compared to women. The number of METhours per week during peak cycling season averaged 40 for the men and 28 for the women. It corresponded to a total energy expenditure of about 3,500 and 1,880 kcal for men and women, respectively. The number of trips per year was about 370, and the annual distance cycled was on average 3,500 km for men and 2,300 for women.

**Conclusion:** Cycle commuting is characterized by equal relative aerobic intensity levels and energy requirements for a given distance cycled by men and women. Based on an overall evaluation, it represents a lower range within the vigorous intensity category. The combined levels of oxygen uptake, durations and trip frequencies lead to high levels of METhours and energy expenditure in both men and women during both peak cycling season as well as over the year. Overall, the study presents a novel basis for interpreting cycle commuting in relation to various health outcomes.

## Introduction

Cycling, together with walking, for transport purposes is one of the more common forms of physical activity. Yet few studies have investigated its basic exercise characteristics, e.g., absolute and relative exercise intensities, MET-values, energy demands, trip durations and frequencies. Knowledge about such variables is of value in itself, and can, when combined, benefit our understanding of the role transport cycling may have on e.g., health outcomes, aerobic capacity and weight control.

Commuting between home and place of work or school is a category of transport trips that is specifically relevant to study since it represents a regular need of transportation during the year (cf. Shephard, 2008). This can be expressed in mean values of between 230 and 280 trips per year, and durations between 14 and 30 min for different groups of cyclists (Stigell and Schantz, 2015). Thus, commuter cycling can be associated with substantial levels of physical activity over the year.

Shephard (2008) wondered whether active commuting could be the answer to population health, and pointed out that “we need a more detailed picture of the typical dose of exercise arising from such activity (the typical duration and intensity of bouts, and number of times performed per week).” Three previous studies have attempted to evaluate the exercise intensities during cycling commuting using heart rate (HR) as an indicator (Kukkonen-Harjula et al., 1988; Oja et al., 1991; Hendriksen et al., 2000), and in one study a portable metabolic instrumentation was used (de Geus et al., 2007). All of those studies involved non-regular cycle commuters. The average exercise intensities reported varied between 55 and 79% of maximal oxygen uptake. This variation creates uncertainty in itself. Moreover, the methodologies used had not been validated in relation to prolonged cycling exercise, neither outdoors nor in a traffic environment. It is, for example, possible that the oxygen pulse differs between laboratory and traffic conditions, which can induce stress (Carroll et al., 2009; Lambiase et al., 2012), as well as over time due to preferential drift of e.g., the HR (Saltin and Stenberg, 1964; Coyle and Gonzalez-Alonso, 2001). Furthermore, portable metabolic devices might not be valid in themselves (cf. Rosdahl et al., 2010), or stable with time passing (cf. Salier Eriksson et al., 2012; Schantz et al., 2018a).

With regard to trip duration, and in particularly in combination with trip frequency, there is a paucity of data (Stigell and Schantz, 2015). It has also been shown that self-reported trip duration with the last figures being 0 and 5, e.g., 15 and 20 min, are associated with systematically higher duration estimates. Furthermore, this type of duration report is highly overrepresented among cycle commuters compared to duration values with the last figures being 1–4 and 6–9 (Schantz, 2017).

To our knowledge, no studies exist of habitual cycle commuting in this respect with validated methods. Against this background, and given our previous validation studies of: (i) a portable metabolic system both in laboratory conditions (Rosdahl et al., 2010) and during sustained outdoor field measurement conditions (Salier Eriksson et al., 2012; Schantz et al., 2018a), (ii) duration reports (Schantz, 2017), and (iii) the creation of a criterion method for active commuting route distances (Schantz and Stigell, 2009), we have considered it important to use these methods to study a number of fundamental exercise physiological and behavioral variables during a normal commute. These include absolute and relative aerobic exercise intensities, ventilation, heart rate responses, MET levels, energy demands, maximal aerobic power, ventilation, ventilatory equivalent, blood lactate, rated perceived exertion, distances, durations and velocities. We have also studied trip frequency per week over the year based on self-reports.

For this purpose we recruited 10 male and 10 female habitual cycle commuters in the metropolitan area of Greater Stockholm, Sweden, and monitored them through laboratory tests as well as during their normal commuting trip with a number of valid or close to valid methods. This enables descriptions of the exercise physiology of cycle commuting in novel ways, and comparisons of the values of men and women. Furthermore, in combining the physiological data with the commuters' frequency of trips per week and over the year as well as their timed trip durations, we create an overall set of data which enable illumination of outcomes in studies within the fields of physical activity and health, exercise and aerobic fitness as well as weight control.

## Materials and Methods

This study is part of a greater multidisciplinary research project, Physically Active Commuting in Greater Stockholm (PACS), at the Swedish School of Sport and Health Sciences, GIH, in Stockholm, Sweden.

### Participants

#### Recruitment of Commuting Participants

The process of selecting participants for this particular study (10 men and 10 women) from a greater sample of cycling and walking commuters was divided into several steps. It started with recruitment through advertisements in the two main morning newspapers in Stockholm in May and June requesting participants. The inclusion criteria required being at least 20 years old; living in the county of Stockholm (excluding the municipality of Norrtälje); and walking or cycling the whole way, any distance to one's work or place of study at least once a year. Answers could be sent in cost-free by post, fax, e-mail or by phone. These advertisements resulted in 2,148 people volunteering to take part.

A paper questionnaire (The Physically Active Commuting in Greater Stockholm Questionnaire 1; PACS Q1) was sent home to these volunteers; 2010 were returned after three reminders via letters through regular mail. The questionnaire consisted of 35 questions but, only the questions relevant for selecting our population were used. These included gender, age, how physically strenuous their professional jobs were as well as commuting frequencies per week for each month of the year and commuting time. The commuting distance of each individual was also used for selecting the study group. These were measured on routes drawn in maps by each respondent. The map measuring method is described in detail in Schantz and Stigell (2009). From the answers from PACS Q1, the respondents were divided into categories based on their reported mode of either cycling or walking or combining both modes. For details on the recruitment and categorization process as well as the questionnaire used, see Stigell (2011).

Our sample was selected from the cyclist category, i.e., those subjects who only cycled to work; (no electrically assisted bicycles were included). Other criteria were that they had ages and route distances close to the median values of the male and female cyclists, respectively (Stigell and Schantz, 2015). They also rated their daily professional jobs as light or very light physically. Letters describing the physiological studies and test procedures were sent to the male and female cyclists who fulfilled the criteria.

The recipients of the letter were first asked if their previously drawn route was still valid, or of a comparable distance time-wise (comparable defined as plus/minus 5–10 min). They then answered a health declaration concerning: (1) medication and for which kind of illness, (2) if they had any palpitations, chest pain or abnormally heavy breathing during exercise, (3) if they had high blood pressure, (4) if they had recently avoided or discontinued exercise for reasons of injury or health. The letter emphasized the right to terminate the tests at any time and without having to stipulate a reason. A signed informed consent of participation was returned.

Based on this information, individuals with invalid route distances as well as with high blood pressure or on medication that could affect normal heart rate were excluded. We contacted the remaining cyclists by telephone to answer any potential questions, and to book test times. Telephone contacts continued until we had 10 women and 10 men who fulfilled the criteria and were willing to participate (Table 1).

**Table 1:** Characteristics of the commuter cyclists (mean and standard deviation, *SD*).

**Cycle commuters**	**Age yrs**	**Height cm**	**Weight kg**	**BMI kg m^**−2**^**	**HR rest beats min^**−1**^**	**Blood pressure, systole mmHg**	**Blood pressure, diastole mmHg**
Men (*n* = 10)	43.6 4.1	185 7.2	85.0 12.7	24.7 3.0	59.1 9.8	123 10	84 8
Women (*n* = 10)	44.0 2.6	170^*^ 5.4	65.8^*^ 7.7	22.6 2.5	60.2 6.7	106^*^ 5	72^*^ 4

* *Significant differences (p < 0.05) between men and women*.

### Equipment and Preparation

#### Stationary Metabolic Gas Analysis System

A stationary metabolic system (Oxycon Pro®, JLAB version 4.53, Carefusion GmbH, Hoechberg, Germany) was used in the mixing chamber mode for all metabolic measurements in the laboratory (SMS). Expired air was sampled continuously from the mixing chamber through a nafion tubing that connects to a nafion tubing on the inside of the equipment that terminates at the oxygen and carbon dioxide analyzer inlets. Ventilation (VE) was measured through a digital volume transducer (DVT) attached to the outlet of the mixing chamber. HR was recorded via a Polar transmitter (see below) and was averaged every 15 s. The equipment was switched on 30 min before data collection and calibrated immediately before and after each test using the built-in automated procedures in accordance with the manufacturer's recommendations. A high precision gas of 15.00% O_2_, and 6.00% CO_2_ (accuracy: O_2_ ± 0.04% rel. and CO_2_ ± 0.1% rel. Air Liquid AB, Kungsängen, Sweden) was used for calibration of the gas analyzers. A facemask with non-rebreathing air inlet valves (Combitox, Dräger Safety, Lübeck, Germany) was used and carefully fitted on the subject. Measured variables were exported to Excel for further processing.

#### Mobile Metabolic Gas Analysis System

A mobile metabolic system (Oxycon Mobile, JLAB version 5.10., CareFusion GmbH, Hoechberg, Germany), complemented with a drying unit for the expired air (see Salier Eriksson et al., 2012), was used for collection of the metabolic variables in the field. This version has been carefully validated and is described in detail elsewhere (Rosdahl et al., 2010; Salier Eriksson et al., 2012; Schantz et al., 2018a). The mobile metabolic system (MMS) was assembled and switched on at a minimum of 30 min before each test. Calibration was performed immediately before and after each experiment using the built-in automated procedures. The “after-calibration” was to control for potential drift during data collection. The calibrations were performed in the same environmental settings as the field measurements. An initial check phase of ~2 min confirmed that a recording was being made, after which the measurement was started. Gas exchange and ventilation variables were measured breath by breath, and averaged over 15 s for further analyses. Two differently sized facemasks (Combitox, Dräger Safety, Lübeck, Germany) were used with the MMS and were carefully fitted on the subject.

#### Ergometer Cycle and Treadmill

A manually braked pendulum ergometer cycle (828E Monark Exercise AB, Vansbro, Sweden) was used. Before each experiment, the scale was zeroed while each subject sat on the saddle with his or her feet resting on the frame between the pedals, and hands resting on the handle bars. The saddle height was adjusted so that the participant's knees were slightly flexed when the feet were on the pedals in their lowest position. The handle bars were adjusted to allow the participants to sit in an upright position. A digital metronome (DM70 Seiko S-Yard Co. Ltd, Tokyo, Japan) helped the subjects maintain the correct cadence while cycling. The workload was controlled every minute by checking the cadence of the participant and the braking force as indicated on the pendulum scale. A treadmill (Model RL2500E, Rodby Innovation AB, Vänge, Hagby, Sweden) was used for measuring the maximal VO_2_.

#### Heart Rate and Blood Pressure

During the exercise protocol, HR was measured and averaged every 15 s using a Polar electro S610i heart rate monitor (Polar Electro Oy, Kempele, Finland) with a Polar Wearlink 31 transmitter (Polar Electro Oy, Kempele, Finland). HR was also recorded and averaged every 15 s with the MMS and the SMS. Blood pressure was measured with a manual sphygmomanometer after 10 min of rest.

#### Blood Lactate Sampling and Analyses

Blood samples (20 μL) for lactate and glucose analysis were taken from a fingertip and immediately transferred to a tube containing haemolysing solution. All samples were analyzed using a BIOSEN 5140 analyzer (EKF-Diagnostik, Barleben/Magdeburg, Germany).

### Quality Controls of the Metabolic Systems

A number of different forms of quality controls of the metabolic systems were undertaken and are described in detail elsewhere (Rosdahl et al., 2010; Salier Eriksson et al., 2012; Schantz et al., 2018a), and will therefore only be briefly mentioned here. For this study it was important to evaluate the comparability of the SMS and MMS, since the SMS measured the VO_2_ in the laboratory and the MMS measured it in the field during cycle commuting. A high level of resemblance between SMS and MMS was noted in the VO_2_ range between 1 and 5 L·min^−1^ (*R*^2^=0.989) (Schantz et al., 2018a). The stability of SMS and MMS during the measurement series was established through checks with a metabolic calibrator (Schantz et al., 2018a). A stability in VO_2_ measurement of the MMS, with a drying unit applied and during stationary field conditions, was noted for a time period of 45 min (Salier Eriksson et al., 2012). The drying unit dries the sample of expired air before it enters into the gas analyzing units of the MMS. Furthermore, external wind was shown not to affect the VO_2_ measurements of MMS (Salier Eriksson et al., 2012). A number of further checks of the validity of VO_2_ measurements of the MMS during the field studies were undertaken, and included the effect of possible alterations in calibration factors. The combined effect of drifts in them amounted to −2.11 ± 2.77% (*n* = 15) for the whole period between the pre- and post-calibrations (Schantz et al., 2018a). Overall, the accumulated evidence from the quality control studies indicate that deviations in MMS from the correct values appear to be small, and of the magnitude of just some few per cent.

### Measurements

The cycle commuters were tested at submaximal and maximal work rates on three different occasions; twice on an ergometer cycle followed by once on a treadmill while the metabolic variables were measured with the SMS. Metabolic measurements were then made during the participants' normal daily commutes using the validated MMS. The reason for repeating the cycle ergometer test protocol twice was to familiarize the participants with the process, and to register if there was any learning effect between the first and second occasion (Schantz et al., 2019). Data from the second cycle ergometer test was used, except for a few cases where the field tests were delayed and a third cycle ergometer test was deemed necessary to ensure valid comparisons between the laboratory and field tests. Two trained investigators carried out the laboratory tests, each participant having the same investigator for each test. Three investigators carried out the field tests; the same investigator always being in charge of the metabolic measurements. The participants were not able to drink during any of the tests because of the mask covering their mouths.

#### Laboratory Tests

Before the laboratory tests, the participants were asked to follow the same standard procedures before each test occasion. These were: (1) not to engage in any vigorous exercise for 24 h beforehand, (2) not to cycle to the laboratory, (3) to refrain from eating, drinking, smoking and taking snuff for at least 1 h before arrival at the laboratory, (4) not to eat a large meal at least 3 h before the tests, (5) to avoid stress, and (6) to cancel the test if they had fever, an infection or a cold. On arrival at the laboratory a check list was ticked off to determine if the participants had followed the standard procedures named above. They were then weighed and measured. A Polar heart rate watch and wearlink were then placed on a wrist and around the chest, respectively. The participants rested quietly on their backs for 10 min on a treatment table. Resting heart rate and blood pressure were determined from the average of the 5 min between the 6 and 10th min.

### Cycle Ergometer and Treadmill Exercise Protocol

Prior to the laboratory tests an evaluation of two test protocols was performed to find the most suitable one for reaching maximal oxygen uptake in normal healthy people and to see if there were any differences between the first and second occasions (cf. Schantz et al., 2019).

The participants cycled at three different work rates, 50, 100 and 150 watt (W) for the women, and 100, 150 and 200 W for the men. A cadence of 50 revolutions per minute (rpm) was chosen (Åstrand, 1952, p. 19). At each work rate the participant cycled until steady-state (~6 minutes), after which the resistance was increased. The third work rate was increased to only 125 W or 175 W for women and men, respectively, if, after the second work rate, the subject's HR was higher than 150 beats·min^−1^ and their perceived rate of exertion (RPE) exceeded 15 for legs and breathing (Borg, 1998, p. 30). The HR from the Polar watch were noted in the protocol after every minute and RPE were noted at the end of each work rate.

Between the second and third work rates the test person continued cycling for 1 min at a self-chosen low cadence with a resistance of 5 Newton. The subject was then asked to resume the cadence of 50 rpm while the investigator slowly increased the work rate until, after 1 min, the third work rate was reached (resistance was increased to 50 W during the first 15 s, to 100 W the second 15 s and successively to the required work rate during the last 30 s). After the sub-maximal test, the subject continued cycling for 2 min at a self-chosen low cadence at 5 Newton.

During the maximal phase, the subjects cycled at a cadence of 80 rpm since it is associated with the longest time to exhaustion in maximal efforts (Foss and Hallén, 2004). For the first three min, the work rates were 60, 100, and 120 or 140 W for 1 min each. The latter alternatives depended on which third work rate the subjects had achieved during the sub-maximal work: 120 W if the third submaximal work rate had been 125 or 175 W for women and men, respectively; 140 W if it had been 150 or 200 W for women and men, respectively. The work rate increased thereafter by 20 W every 60 s. The test continued until exhaustion. HR was noted before each increase of the resistance and also at the moment when the participant terminated the test because of exhaustion.

The treadmill exercise protocol consisted of walking at the speeds 4, 5, 6 km/h with the inclination being 0 degrees until steady state values for HR was attained (normally 5 min), whereafter the speed was increased to an individually comfortable level for running. The inclination of the treadmill was 0 degrees during the first min, 1 degree the second minute, and thereafter raised with 0.5 degrees every minute until exhaustion was reached. Two of the 20 participants did not take part in the maximal treadmill test.

The values for the maximal tests in both ergometer cycling and treadmill running were calculated by averaging the highest consecutive values for VO_2_ and HR over 60 s at maximal exercise. The same 60 s period was used for both VO_2_ and HR.

The Borg scale was used to assess the rated perceived exertion (RPE) (Borg, 1998, p. 30). The subjects were instructed on how to use the scale before commencing the tests. They were asked to point to a number on the scale that corresponded to their feeling of exertion for breathing and in their legs, respectively, before every increase of resistance during the submaximal test and directly after the maximal test. During the maximal phase they continued until exhaustion. To ensure that each subject achieved maximal exertion, at least two of the following three criteria were met by each subject: (1) a plateau in VO_2_ despite increasing power output (defined as a VO_2_ increment of <150 ml), (2) an RER of ≥ 1.1, and (3) a rating of RPE of ≥ 17 on the Borg scale (Borg, 1970; Howley et al., 1995; Midgley et al., 2007).

Due to problems with the MMS which had to be solved and the equipment re-evaluated, 9 to 12 months lapsed before 14 of the field tests took place. Therefore, these participants performed one more rest measurement, as well as sub-maximal and maximal cycle tests in the laboratory, and these values were used as the reference value for the field tests. The mean time between the reference laboratory cycle ergometer test and the field test was 15 ± 10 days.

#### Field Tests During Cycle Commuting

All field test were undertaken between June and November. The participants commuted either to or from their work-place choosing themselves which time was most convenient. Eighteen of the cyclists were tested in the morning rush hours and the remaining two women were tested in the evening rush hours. The field trips took place in the inner urban and suburban areas of Greater Stockholm, Sweden. A detailed description of these areas can be found in Wahlgren and Schantz (2011). The cyclists were met at the designated address by one of the investigators who transported the equipment there by car. The investigator checked that the standard procedures as described above had been followed. The MMS equipment (see preparation of MMS above) was placed in a specially designed backpack on the participant and the gas sampling sensors attached to the previously fitted mask. An initial check phase of ~2 min confirmed that recording was taking place after which the measurement was started. HR was also measured using the Polar electro heart rate recorder. A GPS was placed in the backpack to track the route. This was used for comparisons with the routes drawn on maps by the cyclists. The starting time of the cycle trip was synchronized with the second investigator waiting at the destination. The participants were not able to drink during their cycle commute because of the mask covering their mouths.

On arrival at the destination the cyclists were met by the other investigator. The total trip time was noted, and the cyclist was asked to rate the overall RPE for both breathing and legs. Within 2 min after arrival, blood samples for lactate analyses were taken. The cyclists then stated how many stops they made at traffic lights as well as other stops, and marked them on maps with their routes. They were also asked to confirm whether that route had been taken the whole way, and if not, any deviation from the originally marked route was added in the map. The bicycle was weighed and the type of bicycle was noted.

Oxygen uptake and other measures coupled to the cycling were measured continuously and averaged for every 15 second period from the start to the end of the trip time, and then averaged for the whole time period of the commuting trip.

### Calculations of MET-Values and Energy Expenditure

A MET is here defined as oxygen uptake in ml/kg/min with one MET equal to the oxygen cost of sitting quietly, equivalent to 3.5 ml/kg/min. The average MET-value during each individual's cycle commute was calculated as multiples of the participant's average VO_2_ min^−1^ during the commute divided with the participant's body weight (kg) times 3.5 ml VO_2_ min^−1^ (Balke, 1960). Our rationale for using the definition and values connected to “quietly sitting” is to be able to calculate the additional energy expenditure of commuter cycling compared to the most frequent sedentary position during wake hours, including sitting in cars or in public transit. The caloric coefficients for oxygen used in the energy expenditure calculations were based on non-protein respiratory quotients (Carpenter, 1964, p 104).

### Geographical Area for the Field Tests

The cyclists commuted in the inner urban and/or suburban – rural areas of Greater Stockholm, Sweden. Depending on if they cycled in either one of these areas the routes were classified as “0” or “2,” and with “1” if the routes were partly in both of these areas. The boundary between these areas is described in Wahlgren et al. (2010), and a detailed geographical description of them is given in Wahlgren and Schantz (2011). For the evaluation of this study it is relevant to know that the topography is one of an overall rather flat, but rather gentle slopes of up to normally not more than about 10–15 m height exist. Furthermore, as an indication of residential density of the suburban parts of our study area, we have chosen the southern and westerns suburbs of the Municipality of Stockholm, and in 2005 (a few years before the field tests) this amounted to ~3,500 and 2,900 residents per square km, respectively, whereas in the inner urban area it amounted to 13,000 residents per square km (Municipality of Stockholm, 2008).

### Comparison Between Self-Reported Habitual and Measured Cycling Velocity

Given that de Geus et al. (2007) reported that the cycling velocities of their participants were 10% higher during the days of metabolic measurements than during their habitual commuter cycling, we considered it of value to investigate whether that phenomenon existed in our measurements. We therefore compared the cyclists' velocities, based on the participants' self-reported cycling durations vs. the timed durations. Given that self-reported duration values with the last figure being “0” or “5” represent longer than actual durations (Kelly, 2013; Schantz, 2017), we have, based on the findings of Schantz (2017), compensated for such affects by adding 1.66 km/h to those velocities. Thereafter, paired statistical comparisons of the two attained cycling velocities were undertaken. The mean values were 18.5 ± 2.8 km h^−1^ for the measured cycle velocity, and 18.6 ± 2.5 km h^−1^ for the compensated cycling velocity (n.s.). Thus, we found no indications of differences in cycling velocities during the habitual commuter cycling and the day of the measurement that the cycling durations were timed.

### Ambient Conditions During the Cycling Commuting

The average ambient conditions (temperature, relative humidity and wind speed) during the cycle trip were obtained from the website: The Stockholm-Uppsala Air Quality Management Association (http://www.slb.mf.stockholm.se/lvf/). These are shown in Table 2. There were no significant differences in ambient conditions between the men and the women.

**Table 2:** Ambient conditions during the cycling commuting (mean and *SD*).

	**Temperature (^**°**^C)**	**Relative humidity (%)**	**Wind speed (m s^**−1**^)**
Men (*n* = 10)	10 4	77 12	4.0 2.0
Women (*n* = 10)	12 4	71 22	4.4 1.9

### Statistical Analyses

Statistical analyses were performed using the Statistical Package for the Social Sciences (SPSS, 21.0, Chicago, IL, USA). Results are presented as means and standard deviation (SD) as well as 95% confidence interval (CI) unless otherwise stated. Differences between the men and female values were analyzed with Student's unpaired *t*-tests. Student's paired *t*-test was used to compare cycling velocities at two different occasions. The significance level was set at *p* < 0.05.

## Results

### Responses to Maximal Exercise

Of the measures related to maximal exercise in ergometer cycling, only those connected to maximal oxygen uptake differed significantly between male and female commuters (Table 3). High levels of resemblance were noted between values from maximal ergometer cycling and treadmill running (mean differences ranged +4 and −2%, *n* = 17−18). Only in maximal heart rate was there a significant difference of −2 ± 2% noted for cycling compared to treadmill running (*p* < 0.05).

**Table 3:** Maximal heart rate, oxygen uptake, MET-values and rated perceived exertion (RPE) in maximal ergometer cycling in laboratory (mean, *SD* and 95% CI).

**Cycle commuters**	**Maximal heart rate**	**Maximal oxygen uptake and METmax-values**				**Maximal rated perceived exertion**	
	**beats min^**−1**^**	**L min^**−1**^**	**ml kg bw^**−1**^ min^**−1**^**	**ml kg (bw + cw)^**−1**^ min^**−1**^**	**MET**	**legs**	**breathing**
Men (*n* = 10)	174 7 (169–179)	4.02 0.53 (3.64–4.40)	47.7 5.5 (43.8–51.6)	39.5 3.6 (36.9–42.1)	13.6 1.6 (12.5–14.7)	18.3 1.2 (17.4–19.1)	17.8 1.4 (16.8–18.8)
Women (*n* = 10)	176 11 (168–184)	2.65^*^ 0.37 (2.39–2.92)	40.3^*^ 2.6 (38.4–42.2)	32.3^*^ 2.6 (30.5–34.1)	11.5^*^ 0.7 (11.0–12.1)	17.7 1.5 (16.6–18.8)	18.3 1.5 (17.2–19.4)

* *Significant differences (p < 0.05) between men and women; bw, body weight; cw, bicycle weight*.

### Characteristics of the Cycle Commutes

Characteristics of the cycle commutes are listed in Table 4. The duration, distance and velocity differed between the sexes.

**Table 4:** Characteristics of the commuter cyclists' trips, cycling environments, bicycle weights, number of gears, and stops for different reasons (mean, *SD* and 95% CI).

**Cycle commuters**	**Duration min**	**Distance km**	**Velocity km·h^**−1**^**	**Trips per year**	**Cycling environment**	**Bicycle weights (kg)**	**Number of gears**	**Stops for red lights**	**Other stops**
Men (*n* = 10)	29.1 7.2 (24.0–34.2)	9.61 2.20 (8.05–11.2)	20.1 2.7 (18.2–22.0)	366 146 (262–471)	1.10 0.32 (0.87–1.33)	17.0 2.8 (15.0–18.9)	18.1 7.7 (12.6–23.6)	2.20 1.40 (1.20–3.20)	0.30 0.67 (−0.18–0.78)
Women (*n* = 10)	23.2^*^ 5.0 (19.7–26.8)	6.51^*^ 1.51 (5.43–7.60)	16.8^*^ 1.9 (15.4–18.2)	370 117 (286–454)	0.90 0.57 (0.49–1.31)	16.2 2.3 (14.6–17.9)	14.0 8.7 (7.8–20.2)	2.30 1.77 (1.04–3.56)	0.40 0.97 (−0.29–1.09)

* *p < 0.05. Cycling environment: 0 = inner urban; 1 = inner urban–suburban. For more information about the cycling environments, see Methods under the heading “Geographical area for the field tests”*.

### Oxygen Uptake and MET Values in Cycle Commuting Per Unit of Time

The absolute levels of oxygen uptake per minute in cycle commuting, and the MET values differed between the sexes. The relative intensity of cycle commuting in percent of maximal oxygen uptake did however not differ (Table 5). The added oxygen uptake by the commuter cycling *per se*, i.e. minus the 1 MET that sitting still stands for, did also differ between the sexes (Table 5).

**Table 5:** Oxygen uptake (total and minus 1 MET),% of maximal oxygen uptake, and MET-values in cycle commuting (mean, *SD* and 95% CI).

**Cycle commuters**	**Oxygen uptake in cycle commuting**			**% of maximal oxygen uptake**	**Oxygen uptake of 1 MET**	**MET- values**	**Oxygen uptake in cycle commuting minus 1 MET**		
	**L min^**−1**^**	**mL kg bw^**−1**^ min^**−1**^**	**mL (kg bw + cw)^**−1**^ min^**−1**^**		**L min^**−1**^**		**L min^**−1**^**	**mL kg bw^**−1**^ min^**−1**^**	**mL (kg bw + cw)^**−1**^ min^**−1**^**
Men (*n* = 10)	2.61 0.57 (2.20–3.02)	30.6 4.4 (27.4–33.8)	25.5 4.2 (22.4–28.5)	64.7 10.5 (57.2–72.2)	0.298 0.044 (0.266–0.329)	8.74 1.26 (7.84–9.64)	2.31 0.54 (1.92–2.70)	27.1 4.4 (23.9–30.3)	22.6 4.2 (19.6–25.5)
Women (*n* = 10)	1.70^*^ 0.21 (1.55–1.85)	26.0^*^ 4.0 (23.2–28.9)	20.8^*^ 2.8 (18.8–22.8)	64.7 10.3 (57.4–72.1)	0.230^*^ 0.027 (0.211–0.250)	7.44^*^ 1.14 (6.62–8.25)	1.47^*^ 0.21 (1.32–1.61)	22.5^*^ 4.0 (19.7–25.4)	18.0^*^ 2.8 (16.0–20.0)

* *p < 0.05*.

### Energy Expenditure in Cycle Commuting Per Unit of Time

The absolute levels of energy expenditure in cycle commuting per minute differed between the sexes. The same applies to the levels of energy expenditure minus 1 MET (Table 6). Contrary to that, the respiratory quotients and thereby the caloric coefficients did not differ between the sexes.

**Table 6:** Respiratory exchange ratio, caloric coefficient and energy expenditure (total and minus 1 MET) in cycle commuting (mean, *SD* and 95% CI).

**Cycle commuters**	**Respiratory exchange ratio**	**Caloric coefficient**	**Energy expenditure in cycle commuting**			**Energy expenditure in cycle commuting minus 1 MET**		
	**VCO**_**2**_ $VO_{2}^{-1}$	**kcal VO**_**2**_ **L min**^**−1**^	**kcal min**^**−1**^	**kcal kg bw**^**−1**^ **min**^**−1**^	**kcal (kg bw** **+** **cw)**^**−1**^ **min**^**−1**^	**kcal min**^**−1**^	**kcal kg bw**^**−1**^ **min**^**−1**^	**kcal (kg bw** **+** **cw)**^**−1**^ **min**^**−1**^
Men (*n* = 10)	0.912 0.055 (0.873–0.951)	4.94 0.07 (4.89–4.99)	12.9 2.95 (10.8–15.0)	0.151 0.023 (0.135–0.167)	0.126 0.022 (0.110–0.142)	11.4 2.78 (9.4–13.4)	0.134 0.023 (0.118–0.150)	0.112 0.021 (0.096–0.127)
Women (*n* = 10)	0.930 0.037 (0.903–0.957)	4.96 0.04 (4.93–4.99)	8.42^*^ 1.05 (7.66–9.17)	0.129^*^ 0.019 (0.115–0.143)	0.103^*^ 0.014 (0.093–0.113)	7.28^*^ 1.03 (6.54–8.01)	0.112^*^ 0.019 (0.098–0.126)	0.089^*^ 0.014 (0.079–0.099)

* *p < 0.05*.

### Heart Rate, % of Maximal Heart Rate (%HRmax), % of Heart Rate Reserve (%HRR), Ventilation, Ventilatory Equivalent, Blood Lactate and Rated Perceived Exertion in Cycle Commuting

The absolute and relative levels of heart rate, blood lactate and rated perceived exertion did not differ between the sexes. With regard to ventilation parameters, differences between the sexes were noted in ventilation per minute and tidal volume (Table 7).

**Table 7:** Heart rate, %HRmax, %HRR, ventilation, ventilatory equivalent (VE/VO_2_), blood lactate and rated perceived exertion (RPE) at cycle commuting (mean, *SD* and 95% CI).

**Cycle commuters**	**Heart rate beats min^**−1**^**	**% HRmax**	**% HRR**	**Ventilation (BTPS) L min^**−1**^**	**Ventilatory equivalent**	**Breaths per minute**	**Tidal volume liter**	**Blood lactate mmol Liter^**−1**^**	**RPE leg**	**RPE breathing**
Men (*n* = 10)	136 13 (127–145)	78.3 8.2 (72.4–84.2)	67.2 12.4 (58.3–76.1)	70.8 21.4 (55.5–86.1)	26.8 3.0 (24.6–28.9)	26.9 5,3 (23.2–30.7)	2.63 0,65 (2.17–3.09)	2.87 1.42 (1.85–3.88)	11.5 1.1 (10.7–12.3)	12.8 1.0 (12.1–13.5)
Women (*n* = 10)	137 7 (132–141)	77.7 4.0 (74.8–80.6)	66.2 4.6 (63.0–69.5)	46.6^*^ 6.7 (41.8–51.3)	27.5 2.6 (25.6–29.4)	25.0 4,4 (21.8–28.2)	1.89^*^ 0,25 (1.71–2.06)	2.40 1.29 (1.48–3.32)	11.4 2.5 (9.64–13.3)	12.4 2.0 (11.0–13.8)

* *p < 0.05*.

### Oxygen Uptake and Energy Expenditure in Cycle Commuting Per Unit of Distance

The absolute levels of oxygen uptake and energy expenditure in cycle commuting per km of cycling differed between the sexes, but when dividing those levels with the body weight or together with the cycle weight no differences between the sexes were seen. The same applies to the levels of oxygen uptake and energy expenditure minus 1 MET (Tables 8, 9), i.e., the added oxygen uptake and energy expenditure by the commuter cycling *per se*.

**Table 8:** Oxygen uptake (total and minus 1 MET) per km of cycle commuting (mean, *SD* and 95% CI).

**Cycle commuters**	**Oxygen uptake in cycle commuting**			**Oxygen uptake in cycle commuting minus 1 MET**		
	**L km^**−1**^**	**mL kg bw^**−1**^ km^**−1**^**	**mL (kg bw + cw)^**−1**^ km^**−1**^**	**L km^**−1**^**	**mL kg bw^**−1**^ km^**−1**^**	**mL (kg bw + cw)^**−1**^ km^**−1**^**
Men (*n* = 10)	7.76 1.16 (6.93–8.59)	91.9 11.7 (83.6–100.3)	76.3 8.9 (69.9–82.7)	6.86 1.11 (6.07–7.65)	81.3 11.2 (73.3–89.3)	67.5 8.8 (61.1–73.8)
Women (*n* = 10)	6.10^*^ 0.83 (5.51–6.69)	93.9 16.8 (81.9–105.9)	74.9 11.6 (66.6–83.2)	5.27^*^ 0.79 (4.71–5.84)	81.3 16.0 (69.8–92.7)	64.8 11.2 (56.8–72.8)

* *p < 0.05*.

**Table 9:** Energy expenditure (total and minus 1 MET) per km of cycle commuting (mean, *SD* and 95% CI).

**Cycle commuters**	**Energy expenditure in cycle commuting**			**Energy expenditure in cycle commuting minus 1 MET**		
	**kcal km^**−1**^**	**kcal kg bw^**−1**^ km^**−1**^**	**kcal (kg bw + cw)^**−1**^ km^**−1**^**	**kcal km^**−1**^**	**kcal kg bw^**−1**^ km^**−1**^**	**kcal (kg bw + cw)^**−1**^ km^**−1**^**
Men (*n* = 10)	38.3 6.0 (34.1–42.6)	0.454 0.056 (0.414–0.494)	0.377 0.044 (0.345–0.408)	33.9 5.7 (29.8–38.0)	0.401 0.054 (0.363–0.440)	0.333 0.044 (0.302–0.365)
Women (*n* = 10)	30.3^*^ 4.0 (27.4–33.1)	0.466 0.081 (0.408–0.524)	0.371 0.056 (0.331–0.411)	26.1^*^ 3.8 (23.4–28.9)	0.403 0.077 (0.348–0.458)	0.321 0.054 (0.283–0.360)

* *p < 0.05*.

### METhours and Energy Expenditure in Cycle Commuting Per Week, and the Accumulated Energy Expenditure and Distance Over a Year

The estimated number of METhours and energy expenditure per week during the peak cycling season in the month of May, with a trip frequency per week of 9.5 ± 0.8 for men and 9.6 ± 0.8 for women, as well as values for the accumulated energy expenditure and distance cycled over a year are given in Table 10. The energy expenditure for the mean trip length of 9.61 ± 2.20 km for the men was 372 ± 112 kcal. For the women, the corresponding values were 6.51 ± 1.51 km and 198 ± 59.3 kcal.

**Table 10:** Estimated METminutes, METhours and energy expenditure per week with cycle commuting during the peak cycling season in the month of May as well as the accumulated amount of km cycled and energy consumed during cycle commuting over a year (mean, *SD* and 95% CI).

**Cycle commuters**	**Frequency of trips per week in May**	**METminutes week^**−1**^ in May**	**METhours week^**−1**^ in May**	**kcal week^**−1**^ in May**	**total km year^**−1**^**	**total kcal year^**−1**^**	**(total kcal year^**−1**^) – 1 MET**
Men (*n* = 10)	9.50 0.85 (8.89–10.1)	2,416 747 (1,881–2,950)	40.3 12.5 (31.4–49.2)	3,508 1,017 (2,781–4,235)	3,532 1,742 (2,285–4,778)	133 789 65 480 (86 946–180 632)	118 335 58 605 (76 411–160 258)
Women (*n* = 10)	9.60 0.84 (9.00–10.2)	1,664^*^ 498 (1,308–2,020)	27.7^*^ 8.3 (21.8–33.7)	1,876^*^ 485 (1,529–2,222)	2,320 756 (1,779–2,861)	69 687^*^ 23 550 (52 839–86 534)	60 186^*^ 20 759 (45 336–75 036)

* *p < 0.05*.

## Discussion

This is, to our knowledge, the first study of basic exercise characteristics of habitual cycle commuters using validated methods for all but one variable (declared trip frequency). Furthermore, we combine the physiological and behavioral features in order to create a broader basis for illuminating cycle commuting in relation to the existing literature on morbidity and premature mortality outcomes, aerobic capacity and body weight control. For the first time we also present data on oxygen uptake and energy expenditure, with and without the oxygen uptake of 1 MET, so that the exercise induced changes in these levels as compared to sitting still protrude in a clear fashion.

A main finding is that men and women cycled at the same relative intensity of about 65% of maximal oxygen uptake, despite different levels of cycling durations, distances and velocities (cf. Schantz, 2017). Other important findings are that combining the trip frequency, durations and MET-values led to high values of MET-hours during peak cycling season (40 for men and 28 for women). Furthermore, ventilation was clearly higher than the values used in studies of adverse effects of air pollution for cyclists. Finally, we noted equal levels of energy expenditure per km and kg body weight for men and women although cycling velocities differed.

The findings will be discussed below separately, and in relation to health related variables, physical exercise capacity, and weight control, as well as in relation conceivable methodological advancements.

### Intensity Category

The position stand of the American College of Sport Medicine (ACSM) on the quantity and quality of exercise in relation to sustained health in adults uses both relative and absolute intensities, and describes five different exercise intensity categories: very light, light, moderate, vigorous, near maximal to maximal (Garber et al., 2011). Based on the relative intensity of maximal oxygen uptake, the % HRmax and % HRR, single mode commuter cycling is placed in the bottom – lower part of the vigorous category intensity range (64–90% of VO2 max), whereas the RPE values place it in the light-moderate category [American College of Sports Medicine (ACSM), 1998]. On the other hand, when considering the age of the participants, their MET-values during cycling place them in the upper part of the vigorous category. Blood lactate levels, although not being part of the ACSM-measures, move them again toward the lower intensity group since they were clearly below the 4 mmol levels (cf. Åstrand and Rodahl, 1977, p. 432). The ventilatory equivalents, i.e., levels of ventilation per minute divided with levels of oxygen uptake per minute, were on average about 27 for both sexes. This is indicative of intensities in the transition between the moderate and vigorous intensity domains (cf. Åstrand and Rodahl, 1977, p. 228; Sun et al., 2002). Overall, it seems reasonable to conclude that single mode commuter cycling at about median distances for men and women, respectively, is characterized as being within the bottom – lower part of the vigorous category intensity range.

At the same time it is appropriate to discuss the external validity of this finding. Recent studies indicate that the character of cycle commuting can vary substantially. Double mode active commuters, i.e., those who sometimes cycle and sometimes walk, have shorter cycle distances and lower speeds compared to single mode cyclists (Stigell and Schantz, 2015). Furthermore, commuter cycling velocities are positively related to cycling distances up to rather long distances (Schantz, 2017). This means that one can expect a variation in cycling velocities within both single and double mode cyclist groups of both sexes. Previous studies have shown that cycling speed is related to oxygen uptake (Atkinson et al., 2003; Faria et al., 2005). The present study points in the same direction since, on average, the 20% higher cycling velocities among men, compared to women, are reasonably proportional to higher levels of energy expenditure (-1 MET) per kg body weight (20%) or per kg body and cycle weight (26%) for the men. There are three possible consequences of this. Firstly, that the intensities relative to maximal oxygen uptake increase with cycling distance, secondly that higher levels of maximal oxygen uptake are noted in cyclists that cycle faster, and thirdly, that both the relative oxygen uptakes and the maximal levels of oxygen uptake are higher in commuter cyclists who cycle longer distances. Thus, at present the findings by Schantz (2017) of higher cycling velocities with longer cycling distances must leave us open to both the relative intensity category and the maximal oxygen uptake levels possibly differing for different commuter cyclist groups. This deserves to be studied in future studies.

### MET Values

In epidemiological studies, different types of physical activity are often assigned to different amounts of metabolic equivalents of task (MET). In such cases, they are usually based on a compendium originally published in 1993 (Ainsworth et al., 1993), and updated twice since then (Ainsworth et al., 2000, 2011). In the 2011 version, the MET-value 6.8 is assigned for cycling at the velocities 16.1–19.2 km/h. 17.6 km/h is in the middle of that range. Despite our female cyclists' average velocity being lower than that (16.8 km/h), they had an average of 7.44 MET, i.e., about 10% higher than the 2011 Compendium value. The present male commuter cyclists had a cycling velocity of 20.2 km/h which is in the lower part of the velocity spectrum of 19.3–22.4 km/h, which is assigned 8 MET in the 2011 Compendium. Instead we noted 8.7 MET. Again, our results indicate that for both men and women the relevant MET values should be ~10% higher than the values stated in the 2011 Compendium.

### Energy Expenditure and MET-Hours

According to the ACSM position stand for sustained health, most adults should engage in moderate intensity cardiorespiratory exercise, or in combination with vigorous intensity exercise to achieve a total energy expenditure of ≥8.33–16.7 MET-hours per week (Garber et al., 2011). In a typical week in May, i.e., during the peak of the cycling season (Stigell and Schantz, 2015), the female cycle commuters in our study attained about 28 MET-hours per week, and the men 40. This points to an activity level that is far above the minimal levels, and closer to or even above the optimal levels of health enhancing physical activity (Oja, 2004). Two other examples point in the same direction; Hu et al. (1999) studied the effect of leisure time walking on the risk for type II diabetes in American nurses. A 42% lower incidence of type II diabetes was noted in the highest quintile of walking among the nurses, which corresponded to on average 22 MET-hours per week. Our female cyclists attained 28 MET-hours per week through their cycling (Table 8). Another comparison; in the Harvard alumni study by Paffenbarger et al. (1986), an energy turnover of leisure time levels of physical activity of 3,000–3,500 kcal per week led to a maximal reduction in all-cause mortality of 54%, a level that our male cyclists reached during peak cycling season (Table 8). Matthews et al. (2007) noted a 34% decrease in premature mortality in Chinese women with more than 3.4 MET-hours/day of commuter cycling. Our female cyclists reached 5.5 MET-hours/day. Matthews et al. (2007) assigned 4 METs for cycling in Shanghai, China, whereas we measured it to be 7.44 in Stockholm, Sweden. Even when considering these differences the Stockholm female cyclists should be able to gain about the same reduction in premature mortality as the female cyclists did in Shanghai. This example points to the importance of selecting a relevant MET-value, and basing it on empirical data for the cycling behavior of the group being studied. Otherwise, errors are introduced in our attempts to describe relevant dose-response relations of e.g., health outcomes.

### Ventilation

Levels of ventilation are related to intensity of cycling, and are the focus of a number of studies given that inhalation of various forms of air pollution can lead to negative impacts on cyclists' health (cf. de Hartog et al., 2010; Raza et al., 2018). The most developed tool for health impact assessments of cycling (WHO, 2017) takes into account negative effects of air pollution. The ventilation in cyclists in that tool is set to 2.55 m^3^/h, whereas our findings indicate higher levels; means for men; 4.2 m^3^/h and 2.8 m^3^/h for women. Bigazzi and Figliozzi (2014) in their review of intake and uptake of traffic-related air pollution also base the intake on lower values than the ones we attained. A number of their values are based on ventilation levels of cyclists that are only about double those of car drivers, which in our minds are unrealistically low values, unless very low and unlikely cycling speeds are applied. Overall these matters need to be pursued with studies using validated measurement tools of ventilation and reasonable cycling intensities (cf. Raza et al., 2018).

### Maximal Aerobic Capacity and Maximal Exercise Capacity

The maximal oxygen uptake levels can be about 5–10% higher if maximal exercise is performed on a treadmill compared to a cycle ergometer (cf. Åstrand and Rodahl, 1977, p. 335). The consequence of this is that a certain submaximal oxygen uptake will stand for different levels relative to the maximal oxygen uptake, and thereby differ between different studies merely due to differing absolute reference points. We did, however, not notice any differences in maximal oxygen uptake in this respect in the cycle commuters, which is in line with findings on competitive cyclists (Basset and Boulay, 2000). Furthermore, support for that the levels measured represent the participants' levels of maximal oxygen uptake comes from a reproducibility study of the same participants displaying a high level of concordance between test and retest (Schantz et al., 2019).

Aerobic capacity can be divided into two dimensions; (i) the maximal capacity of oxygen uptake, and (ii) the ability to make use of a larger portion of that during prolonged exercise durations (Åstrand and Rodahl, 1986, p. 421). Both of these aspects limit the maximal physical work capacity. However, the latter capacity is also dependent on muscle mass and function as well as anaerobic capacity. Of these capacities, it appears that the maximal oxygen uptake, in itself, may be linked to health outcomes (Ross et al., 2016; Al-Mallah et al., 2018; Tikkanen et al., 2018; Kandola et al., 2019).

The general knowledge is that exercise related increases in maximal oxygen uptake depends on training status, intensity, duration, and frequency, and that these aspects can modify the effect level, independently of each other. If one is untrained, exercise intensities from 50% of max VO2 and more induce increases in aerobic capacity (cf. Wenger and Bell, 1986). But if one is somewhat aerobically trained, exercise intensities of 50% of max VO2 will not be sufficient to increase the maximal oxygen uptake even if the exercise is undertaken 6 h a day, 6 days per week for 2 months (Schantz, 1980; Schantz et al., 1983). However, at the same time, a greater fraction of the maximal oxygen uptake can be used after prolonged training at such exercise intensities (Schantz, 1980). Thus, it seems that adaptations in these two outcomes can vary independently of each other.

Physical training appears to be able to increase maximal oxygen uptake in all previously non-trained individuals if only duration, intensity and frequency are sufficient (Montero and Lundby, 2017). Will then the present findings of exercise intensities of, on average, 65% of maximal oxygen uptake, and average durations of 20–30 min repeated about 9.5 times per week lead to increased maximal oxygen uptake? An indication that this might be the case is that the estimated maximal oxygen uptake levels in our samples were on average about 31–39% higher than the average age-matched values in the general population (Schantz et al., 2018b). However, those differences could also be due to selection factors.

Support for the higher maximal oxygen uptake levels possibly being partly due to training effects from the commuter cycling comes from the ACSM standpoint [American College of Sports Medicine (ACSM), 2000] that exercise HR in the range of 60–90% of HRmax and 50–85% of HRR is effective for aerobic fitness training. The mean values for the cycle commuters in this respect was 78% of max HR and 67% of HRR. However, normally the intensities used are tightly controlled and fixed in the type of training studies that constitute the bases for most of our understanding of aerobic training effects (cf. Wenger and Bell, 1986), whereas the intensities fluctuate during commuter cycling in free-living conditions. This creates uncertainties about the external validity of results from studies of continuous exercise to exercise with fluctuating intensities.

There are few longitudinal studies of effects of commuter cycling in adults and on the maximal aerobic capacity, and the ones that exist are all based on more or less untrained participants. In general their findings support that rather modest increases in maximal oxygen uptake (6–8%) may occur (Oja et al., 1991; Hendriksen et al., 2000). However, that can be due to modest levels of trip frequencies and/or durations. Notably, physiological responses in terms of maximal work capacity follow a dose-response relationship, in which the initial fitness levels are also important (Hendriksen et al., 2000). A final comment relating to aerobic capacity is that with commuter cycling, the perceived exertion decreased at a standard physiological strain of 85% of the maximal oxygen uptake (Oja et al., 1991). Although it was not measured, this could lead to an ability to make use of a greater portion of the maximal oxygen uptake during cycling. There is a great need to further our understanding of these issues in future studies.

### Weight Control and Supply of Nutritients

There is anecdotal evidence that physicians in Stockholm, Sweden, already during the late 18th century, ordinated physical activity to try to diminish obesity (Schantz, 2014), and since the evolvement of the industrial era in Europe during the 19th century, the issues of both overweight and obesity have attracted increasing attention [e.g., Banting (1870)]. During the last three decades, an unexpected epidemic of increasing body weights [e.g., Jaacks et al. (2019)], with concomitant health problems of various sorts, have led to a great interest as to what extent physical activity behaviors can counteract this development. At the same time, less focus has been on the value of physical activity in increasing energy turnover and thereby facilitating the supply of essential nutrients to the body through an increased food intake (Åstrand and Rodahl, 1986, p. 574–575; Heydenreich et al., 2017). Both these dimensions will be discussed below.

Evidence is accumulating that cycle commuting may play a positive role in relation to overweight and weight control. For example, Lusk et al. (2010) showed a decrease in body weight with increased cycling, as well as vice versa. Flint et al. (2016) performed a cross-sectional study using UK Biobank data, and showed that active commuters are associated with a lower BMI and percentage of body fat compared to passive commuters and mixed public transport and active commuters. Mytton et al. (2018) noted relationships between active commuting and less visceral adipose tissue in a cross-sectional study. Quist et al. (2018) reported a meaningful reduction in adipose tissue in obese and over-weight men and women who cycle commuted in a randomized control study.

The present study provides data on energy demands of cycle commuting which are valuable for interpreting cycle commuting in these contexts. Based on the energy content in 1 kg fat tissue that is about 6,000–7,000 kcal (Åstrand and Rodahl, 1977, p. 485), the estimated average overall energy expenditure in male cycle commuters corresponded to about 17–20 kg fat tissue per year, after subtracting the energy levels of 1 MET from sitting still. The corresponding value for women was about 9–10 kg fat tissue per year. Based on Ross and Janssen (2001), weekly body weight losses of about 0.4–0.6 kg could be anticipated with the weekly energy expenditures from commuter cycling described in this study, and for a time period of at least up to 4 months. After that, and with time passing, it seems that weight reduction diminishes and is more difficult to achieve through physical activity (Ross and Janssen, 2001). Finally, given the cycle commuters' weekly energy expenditures through cycling during peak cycling season of about 1,900 kcal for the women and 3,500 kcal for the men, there is no doubt that cycle commuting can be valuable in transforming low energy consumers (1,500–2,000 kcal/day) into high ones (2,500–3,000 kcal/day), and in that way facilitate an intake of sufficient levels of essential nutrients (Åstrand and Rodahl, 1986, p. 574–575; Heydenreich et al., 2017).

### Energy Expenditure Per Distance

An important finding was that the overall energy cost for a given distance of cycling is the same for men and women despite different cycling speeds (0.46 kcal per kg body weight and km). As far as we know, Leo Zuntz pioneered systematic studies of oxygen uptake and energy demands during cycling (Zuntz, 1899). Based on distinct energy values from measurements on one individual weighing 71 kg, in the cycling speed range between 8.8 and 21.1 km/h (cf. Passmore and Durnin, 1955), the energy expenditure per km and kg body weight was very stable at about 0.423 kcal per kg body weight and km. That is very close to the values in this study. Thus, interestingly these studies jointly speak in favor of a stability in energy expenditure for a given distance independent of cycling velocity. This is worth investigating in future studies. One important reason for this is that it indicates a possibility to make use of this measure as a proxy for both the duration and the intensity measures. As stated above, it is not easy to obtain valid data on these two important measures (Schantz, 2017; Schantz et al., 2018a). In contrast to this, both body weight and route distances, based on GIS and origin as well as destination points or GPS-tracings in e.g., smart phones, are relatively easy to gather, thus standing out as an interesting methodological option to use in future epidemiological studies of cycling. This deserves therefore to be further studied and hopefully confirmed as a methodological advancement.

Finally, the oxygen cost of about 90 ml per km and kg body weight in our study, points to about 15–40% lower energy cost during cycling than during walking at different speeds, and about 55% lower oxygen cost than during running at different velocities (Saibene and Minetti, 2003).

### Strengths and Limitations

A great strength of this study is that we base it on meticulous methodological studies in relation to metabolic measurements in the laboratory and in the field (Rosdahl et al., 2010; Salier Eriksson et al., 2012; Schantz et al., 2018a). Likewise, route distances were measured with a criterion method (Schantz and Stigell, 2009), cycling durations were timed, body weights as well as bicycle weights were measured, and the number of stops were counted. Another strength was that the studied individuals in several respects were representative for single mode cyclists (cf. Stigell and Schantz, 2015). The primary limitations include the low number of men and women studied, and that we have not studied double-mode cyclists. Furthermore, the self-reported frequency of trips has not been validated.

Considerable efforts were undertaken to ensure that the metabolic measurement methods used were valid over the time that the study was undertaken, both in the laboratory and under sustained field conditions with the climatological conditions prevailing during large parts of the year in Stockholm, Sweden, as well as for relevant levels of metabolism during cycle commuting (Rosdahl et al., 2010; Salier Eriksson et al., 2012; Schantz et al., 2018a). In only one variable, the respiratory exchange ratio, was a significant difference of some percent noted between the mobile measurement system (Oxycon Mobile) and the criterion method (the Douglas Bag Method) (Rosdahl et al., 2010). The effect of this on the caloric coefficient is however negligible, and is well-within the limits of uncertainty of some percent in overall levels of metabolism during cycle commuting that we have reported elsewhere (Schantz et al., 2018a).

While the measurement equipment is one part of the validity dimension, the characteristics and behaviors of the participants is another equally important dimension to consider. Our participants were habitual commuter cyclists who were selected from individuals in a greater sample with characteristics close to the mean ages and the median values for cycling distances as well as speed differences between the sexes (cf. Stigell and Schantz, 2015). Furthermore, as described in Methods, their estimated habitual cycling velocities did not differ from those during the measurement day for this study. Furthermore, men and women cycled in comparable ambient conditions, including wind (cf. Table 2). Finally, their average cycling velocities closely resembled those of both sexes in a greater sample of commuter cyclists (Schantz et al., 2018b).

Final methodological remarks relate to the external validity. The landscape cycled in is overall rather a flat landscape, but some gentle slopes of up to about 10–15 m height exist (see Methods). The traffic landscape is in the metropolitan region of Greater Stockholm, with an inner urban densely built-up area and suburban as well as a rural areas. In general, cycling velocities were affected by where one cycles, but the overall effect is rather small. Inner urban cycling velocities are on average 0.65 km/h lower than in suburban–rural areas in Greater Stockholm (Schantz, 2017). The average cycling area in this study was a combination of inner urban and suburban-rural areas for both men and women (cf. Table 3). Part of the reason for the area difference in cycling velocities is most likely varying numbers of stops for red lights or other reasons, and that did not differ between the sexes.

A final remark coupled to external validity relates to commuter cyclists not being a homogenous group. We have identified one subgroup termed single mode cyclists who never walk to their places of work or study, and another subgroup of dual modality cyclists, who sometimes walk and sometime cycle (Stigell and Schantz, 2015). Our study is limited to single mode cyclists and comments on the possible consequences of this regarding external validity have been stated above.

## Conclusion

In conclusion, cycle commuting in single mode cyclists is characterized by equal relative aerobic intensity levels for men and women. The same applies to energy requirements for a given distance cycled when taking body weight differences into account. Based on the MET-values, it can be classified as being in the upper part of vigorous physical activity. However, the relative oxygen uptake intensities,% of HRR,% of HR max, blood lactate levels and rated perceived exertions favor a conclusion that cycle commuting represents a low intensity range within the vigorous category. Ventilation during cycling was clearly higher than the values normally used in studies focusing on possible adverse effects of cycling in air pollution. The high levels of MET-hours noted per week for both men and women during peak cycling season, and the yearly levels of distance cycled and energy consumed, point to substantial positive effects on various health outcomes, aerobic fitness, maximal exercise capacity, weight reduction, weight control, and facilitating the supply of essential nutrients through food intake.

## Data Availability Statement

The datasets generated for this study are available on request to the corresponding author.

## Ethics Statement

The studies involving human participants were reviewed and approved by Ethics Committee North of the Karolinska Institute at the Karolinska Hospital (Dnr 03-637), Stockholm, Sweden. The patients/participants provided their written informed consent to participate in this study.

## Author Contributions

PS conceived the overall aim of the study and designed it. JS, HR, and PS were responsible for collecting and analyzing the data. HR was responsible for the quality of the measurement devices in the laboratory. JS drafted the first version of the method section. PS drafted the remainder of the manuscript. All authors read, commented, and accepted the final manuscript.

## Conflict of Interest

The authors declare that the research was conducted in the absence of any commercial or financial relationships that could be construed as a potential conflict of interest.
